# The Relationship between Red Blood Cell Distribution Width and Incident Diabetes in Chinese Adults: A Cohort Study

**DOI:** 10.1155/2020/1623247

**Published:** 2020-02-27

**Authors:** Jialu Wang, Yanan Zhang, Yanping Wan, Zhuping Fan, Renying Xu

**Affiliations:** ^1^Department of Clinical Nutrition, Ren Ji Hospital, School of Medicine, Shanghai Jiao Tong University, Shanghai, China; ^2^Department of Gastroenterology, Ren Ji Hospital, School of Medicine, Shanghai Jiao Tong University, Shanghai, China

## Abstract

**Background:**

Previous studies reported the controvertible association between red blood cell distribution width (RDW) and diabetes. The aim of this study is to explore whether RDW is associated with incident diabetes.

**Methods:**

We performed this cohort study in 16,971 Chinese adults (9,956 men and 7,015 women, aged 43.3 ± 12.8 years). The level of RDW was measured at baseline (2014). All the participants were further classified into four quartile groups based on baseline RDW. Fasting blood glucose (FBG) and glycated hemoglobin A1c (HbA1c) were measured annually during follow-up (2014-2019). Diabetes was diagnosed if either FBG ≥ 7.0 mmol/L or HbA1c ≥ 6.5%. We used the Cox proportional hazards regression model to evaluate the association between baseline RDW and incident diabetes.

**Results:**

We identified 2,703 new cases of diabetes during five-year follow-up. The incidence was 15.9%. Comparing with participants in the lowest quartile group (reference group), the adjusted hazard ratios (HR) for the risk of diabetes were 1.31 (95% CI: 1.16, 1.48) for the highest quartile group (*p* trend < 0.001), after adjustment for potential confounders. Further adjusting baseline FBG and HbA1c did not materially change the association between RDW and incident diabetes. Each unit increase of RDW was associated with a 16% higher risk of incident diabetes (HR = 1.16, 95% CI: 1.06, 1.26) in a fully adjusted model. Sensitivity analysis generated similar results with prospective analyses after excluding aged participants, participants who are overweight and with obesity, participants with elevated blood pressure, participants with decreased eGFR, and those with anemia at baseline.

**Conclusions:**

High RDW was associated with high risk of developing diabetes in Chinese adults. As RDW is an inexpensive, noninvasive, and convenient indicator, RDW might be considered for inclusion in the risk assessment of high-risk groups of diabetes.

## 1. Introduction

The number of people with diabetes has doubled during the past 20 years worldwide [1]. The global prevalence of diabetes in adults is estimated to be 8.8% in 2015 and 10.4% in 2040, as reported by the International Diabetes Federation (IDF) [2]. People with diabetes are more likely to develop cardiovascular disease than those nondiabetic individuals [3], thus throwing huge burden to both families and the society. It is meaningful to implement early intervention to those participants with high risk of diabetes, thus curbing the increasing trend of diabetes and decreasing the risk of cardiovascular disease [4].

Red blood cell distribution width (RDW) is a hematological parameter, which reflects the heterogeneity of erythrocyte volume, and it is traditionally used, along with the mean corpuscular volume, to tell the subtypes of anemia [5]. In recent years, several studies had assessed the possible association between RDW and diabetes [6–11]. However, studies in diabetic population generated inconsistent results. One cross-sectional study reported a significantly positive association between RDW and glycated hemoglobin A1c (HbA1c), independent of fasting blood glucose (FBG) levels in nondiabetic American adults [6] while the other reported a negative correlation between RDW and fasting glucose in elderly Chinese [7]. RDW was significantly higher in diabetic patients than healthy subjects and particularly higher in uncontrolled glycemia in a retrospective case-control study [8] while the other one [9] found that type 2 diabetes patients with higher RDW had significantly lower risk of poor glycemic control. The results of cohort studies were also inconsistent [10, 11], due to sample size and the level of adjustment. Cohort studies with large sample size are limited.

Thus, we perform the current cohort study to evaluate the association between RDW and the risk of incident diabetes in about 17,000 Chinese adults during five-year follow-up. A series of conventional risk factors for diabetes, including age, sex, BMI, blood pressure, and lipid profiles, were taken into account.

## 2. Materials and Methods

### 2.1. Study Population

**Figure 1:**
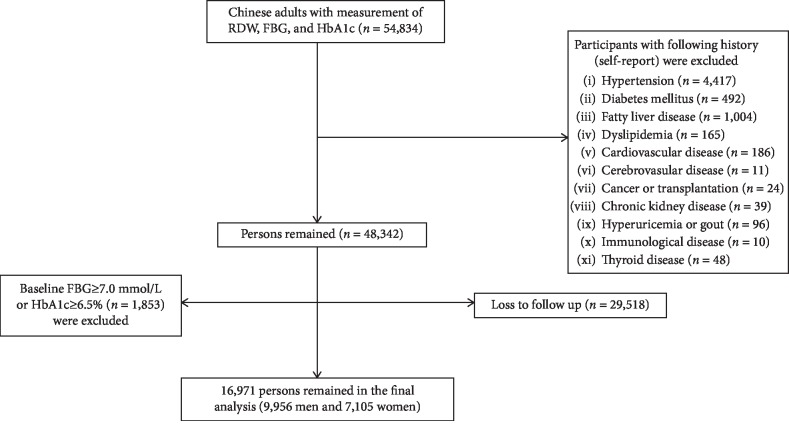
Flow chart of sample recruitment. Abbreviation: RDW: red blood cell distribution width; FBG: fasting blood glucose; HbA1c: glycated hemoglobin A1c.

All the participants (≥18 y) were recruited from communities and have taken a healthy checkup at Health Management Center, Ren Ji Hospital, from January 1, 2014, to May 31, 2019. A total number of 53,834 Chinese adults were eligible for the study. The level of RDW was measured at baseline (2014). FBG and HbA1c were measured annually during follow-up (2014-2019). We performed a sequential process of sample recruitment. Because incident diabetes is strongly associated with the history of hypertension and diabetes, we first excluded participants with hypertension (*n* = 2,101), with diabetes (*n* = 492), and with both of them (*n* = 2,316). Then, we excluded participants with other chronic diseases (*n* = 1,355). Further, we excluded participants with cancer, chronic kidney disease, immunological disease, and thyroid disease because these diseases might associate with RDW. Finally, we excluded participants whose FBG ≥ 7.0 mmol/L or HbA1c ≥ 6.5 mmol/L at baseline (*n* = 1,853) and those lost to follow-up (*n* = 29,518); a total number of 16,971 Chinese adults (9,956 men and 7,015 women, aged 43.3 ± 12.8 years) were included in the study (Figure 1). Taken together, we excluded 4,661 participants (8.5%) due to prevalent diabetes. Compared with those out of the study, the participants included in the study were younger, with lower level of FBG and HbA1c and with similar level of RDW and proportion of women (Supplementary [Supplementary-material supplementary-material-1]). The study protocol was approved by the Ethical Committee of Ren Ji Hospital, School of Medicine, Shanghai Jiao Tong University.

### 2.2. Assessment of Diabetes, RDW, and Other Biochemical Parameters

All the measurements were completed in the Clinical Laboratory of Ren Ji Hospital. Venous blood samples were drawn and transfused into vacuum tubes containing EDTA in the morning after participants were fasted overnight for eight hours. The RDW in the current study refers to RDW-CV (red blood cell distribution width-coefficient of variation). The level of RDW, together with red blood cell count, white blood cell count, hemoglobin, and hematocrit, was measured by an automatic hematology analyzer (XN-10, Sysmex, Japan). All the participants were further classified into four quartile groups based on baseline RDW.

FBG was measured by enzyme-linked immunosorbent assay (Roche 701 Bioanalyzer, Roche, UK). HbA1c were measured by a high-performance liquid chromatography method (variant II automatic glycosylated hemoglobin analyzer, Bio-Rad, America). Diabetes was confirmed if either FBG ≥ 7.0 mmol/L or HbA1c ≥ 6.5% [12].

Alanine transferase, aspartate transferase, total cholesterol, triglycerides, high-density lipoprotein cholesterol, low-density lipoprotein cholesterol, and high-sensitivity C-reactive protein were measured as well. The estimated glomerular filtration rate (eGFR) was calculated using the Chronic Kidney Disease Epidemiology Collaboration 2-level race equation [13].

### 2.3. Assessment of Other Confounders

Body weight and height were measured in light clothes with no shoes at baseline, and BMI was calculated by body weight in kilograms divided by square of height in meters. Overweight (24.0 ≤ BMI < 28.0 kg/m^2^) and obese (≥28.0 kg/m^2^) were confirmed based on BMI cutoff points for Chinese adults [14]. Blood pressure was measured twice using an automatic blood pressure meter (HBP-9020, OMRON (China) Co., Ltd.) after participants were seated for at least 10 min. The average of two measurements was recorded for further analysis.

The history of hypertension, diabetes, fatty liver disease, hyperlipidemia, cardiovascular disease, cerebrovascular disease and stent surgery, cancer/transplantation, chronic kidney disease, hyperuricemia/gout, immunological disease, and thyroid disease was collected via a self-report questionnaire.

### 2.4. Statistical Analysis

We completed all statistical analyses by SAS version 9.4 (SAS Institute, Inc., Cary, NC). Formal hypothesis testing will be two-sided with a significant level of 0.05.

In the current study, we used the Cox proportional hazards regression model to evaluate the association between RDW and incident diabetes. The person-time of follow-up for each participant was determined from the baseline to (January 1, 2014) to either the onset date of diabetes, loss to follow-up, or the end of follow-up (May 31, 2019), whichever came first.

We adjusted for potential confounders in different models: model 1, adjusting for age (y) and sex; model 2, adjusting for variables in model 1, and further BMI (kg/m^2^), systolic blood pressure (mmHg), diastolic blood pressure (mmHg), total cholesterol (mmol/L), triglycerides (mmol/L), low-density cholesterol lipoprotein (mmol/L), high-density cholesterol lipoprotein (mmol/L), eGFR (mL/min per 1.73 m^2^), alanine transferase (U/L), and aspartate transferase (U/L) at baseline; model 3, adjusting for variables in model 2, and further hemoglobin (g/L), hematocrit(10^9^/L), red blood cell (10^12^/L), and white blood cell (10^9^/L); model 4, adjusting variables in model 3 and high-sensitivity C-reactive protein (mg/L) to further determine whether the association between RDW and incident diabetes is driven by systemic inflammation; Finally, we further adjusted baseline FBG (mmol/L) and HbA1c level to determine whether baseline level had effects on the risk of incident diabetes although we realized that it might be at risk of overadjustment.

We tested the interaction between of age and sex with RDW, in relation to incident diabetes. To test the robustness of the results obtained from the main analysis, we conducted five sensitivity analyses: excluding elder participant (≥65 y), overweight and with obesity [14], with elevated blood pressure (systolic blood pressure ≥ 130 mmHg or diastolic blood pressure ≥ 80 mmHg) [15], with decreased eGFR (≤60 mL/min per 1.73 m^2^) [13], and with anemia (male: hemoglobin < 120 g/L, female: hemoglobin < 110 g/L) [16], respectively.

## 3. Results

The mean age, FBG, and HbA1c were 43.3 ± 12.8 y, 5.0 ± 0.5 mmol/L, 5.3 ± 0.3%, respectively, while the mean RDW was 12.8 ± 0.9%. RDW was associated with all the characteristics at baseline (Table 1).

During five years of follow-up, we identified 2,703 new cases of diabetes. The incidence was 15.9% (36.2/1000 person-years). Comparing with participants in the Q1 group, the adjusted hazard ratios (HR) for the risk of diabetes was 1.04 (95% CI: 0.91, 1.17) for the Q2 group, 1.17 (95% CI: 1.04, 1.32) for the Q3 group, and 1.31 (95% CI: 1.16, 1.48) for the Q4 group (*p* trend < 0.001), after adjusting a series of potential confounders (Table 2, model 4). Further adjustment of baseline FBG and HbA1c did not materially change the association between RDW and diabetes. Each unit increase of RDW was associated with a 16% higher risk of incident diabetes (HR = 1.16, 95% CI: 1.06, 1.26) in fully adjusted model (Table 2, model 5). RDW was also associated with impaired fasting glucose (defined as FBG ranging from 6.1 mmol/L to 7.0 mmol/L). Each unit increase of RDW was associated with an 11% higher risk of incident diabetes (HR = 1.11, 95% CI: 1.04, 1.19, *p* trend = 0.004) after adjusting the same variables in model 5. We have further conducted the analysis which included those excluded participants and adjusted for the metabolic conditions in the model and got similar results (HR = 1.2 (95% CI: 1.11, 1.3) for each percent increase in RDW). The details are shown in Supplemental [Supplementary-material supplementary-material-1].

We did not find the interaction between age and sex with baseline RDW, in relation to the risk of diabetes (both *p* > 0.05) (Supplementary [Supplementary-material supplementary-material-1]). Excluding elder participants (≥65 y), participants who are overweight and with obesity, with elevated blood pressure, with decreased eGFR (<60 mL/min/1.73m^2^), and those with anemia at baseline, similar results were generated with the prospective analyses (Table 3).

## 4. Discussion

In the current cohort study including 16,971 Chinese adults, we found that a higher level of RDW was associated with higher risk of incident diabetes, after deliberately adjusting conventional risk factors for diabetes, a series of hematological index, high sensitivity C-reactive protein, FBG, and HbA1c.

We identified 2,703 new cases of diabetes during five years of follow-up. The incidence of diabetes in the current study was 15.9% (36.2/1000 person-years). Data from the Shanghai Women's Health Study (SWHS) reported that the incidence of diabetes was 7.13/1000 person-years [17]. However, the study was performed decades ago. National surveys showed that the prevalence of diabetes has increased dramatically (≈17-folds) in the past several decades in Mainland China [18]. Further, all the participants in the SWHS were women while 60% of the participants were men in our study. The prevalence of diabetes was higher in men than that in women, which might be another possible reason for the differences between the SWHS and our study. The incidence of diabetes was also higher than another cohort study in China [19], which reported that the incidence of diabetes was 11.08/1000 person-years based on the definition by FBG ≥ 7.0 mmol/L, and/or the use of hypoglycemic drug, and/or diagnosed medical history of diabetes. However, it was comparable with the data from Shanghai Municipal Center for Disease Control and Prevention. They reported that the overall weighted prevalence of diabetes was 17.6% (95% CI: 16.4%-18.8%) among 18736 adults [20]. An estimated prevalence of diabetes in China was 11.6% based on similar definition of diabetes (either FBG or HbA1c); however, the author pointed out that 8.1% of the participants with diabetes might be underdiagnosed diabetes [21]. However, we cannot exclude the possibility of misclassification of diabetes, because participants confirmed with diabetes had not been further assessed by oral glucose tolerance test (OGTT).

Previous studies on the relationship between RDW and diabetes only examined the relationship between RDW and fasting glucose or glycosylated hemoglobin, and most of them were cross-sectional studies. A cross-sectional study which included 15,343 nondiabetic adults (enrolled in NHANES 1999–2008), free of cardiovascular diseases, firstly reported a significantly positive association between RDW and HbA1c, independent of FBG levels [6]. Another new published survey of the relationship between RDW and metabolic syndrome in elderly Chinese showed that RDW demonstrated positive correlations with age and systolic blood pressure but negative correlations with triglycerides and fasting glucose [7]. Although these two cross-sectional studies found the connection between RDW and HbA1c or FBG, they were limited by the inherent shortcomings of cross-sectional design. Case-control study generated inconsistent results. RDW was significantly higher in diabetic patients than that in healthy subjects and was particularly higher in uncontrolled glycemia in a retrospective case-control study [8] while the other [9] demonstrated type 2 diabetes patients with higher RDW had significantly lower risk of poor glycemic control. Few cohort studies have been conducted and the conclusions are also inconsistent. A retrospective cohort study, including 2,688 individuals (aged 49-66 years) without diabetes, impaired fasting glucose, or anemia at baseline, showed that high RDW is associated with the high risk of incident diabetes over 3-4-year follow-up in middle-aged and older Chinese adults [10]. In contrast, another cohort study reported that low RDW is associated with increased incidence of diabetes in 2,944 participants over 14-year follow-up [11]. The reasons for the inconsistent conclusions may lie in sample size and failure to adjust confounding factors. Therefore, we expanded the sample size and take most of conventional risk factors into consideration. As in the current study, we found that high RDW was associated with high risk of developing diabetes in Chinese adults.

The underlying mechanism between RDW and diabetes could be explained by several pathways. Hyperglycemia leads to changes in red blood cells, resulting in changes in erythrocyte structure and hemodynamic characteristics [22, 23]. Further, hyperglycemia has effects on the lifespan of red blood cells and contributed to a high variability in red blood cell volume [24]. Several proinflammatory cytokines could inhibit synthesis or activity of erythropoietin [25] and diabetes has been considered a proinflammatory state [26]. Both abnormal erythropoietin production and erythropoietin hyporesponsiveness might induce a gradual increase in RDW values [27, 28]. Finally, oxidative stress has a profound influence on erythrocyte homeostasis and survival [29], thus leading to the increase of RDW.

The strengths of our study included cohort study design, taking most of potential confounders into consideration and large sample size. However, some limitations need to be addressed. First, we did not know the daily intake of iron, folic acid, and vitamin B_12_ levels. Thus, we excluded participants with anemia and adjusting red blood cell, white blood cell, hematocrit, and hemoglobin in the model; this might alleviate the potential distractions. Second, behavior information such as alcohol intake, smoking habit, and exercise was deficient, and these factors were closely associated with incident diabetes. Third, we did not collect information on antidiabetes medications during follow-up, which could result in the loss of new diabetes case. Further, diabetes was confirmed by either FBG or HbA1c but had not further been assessed by OGTT, which might lead to misclassification of diabetes status. However, it was very difficult to apply oral glucose tolerance test in an epidemiological study with large sample size [30]. It is true that we excluded patients with self-reported diabetes in the sequential recruitment of study population, and we cannot exclude the possibility that some of the participants with diabetes were still included in the study due to undiagnosed diabetes [21] and recall bias. Finally, more than half of the participants were lost to follow-up because they changed the checkup hospital or did not perform health checkup again after baseline survey. However, about 17,000 participants remained in the study, and it was still a big sample size compared with previous studies [10, 11]. Another limitation was that only about 40% of the participants were women. We have tested the interaction of sex with the relationship between RDW and incident diabetes; it seemed that there were no obvious differences between men and women. However, we could not determine the causal relationship between RDW and diabetes. Further prospective studies with large sample size are needed to confirm our results.

## 5. Conclusion

High RDW was associated with high risk of developing diabetes in Chinese population. Despite the shortcomings of our study, our five-year cohort study clearly concluded that the risk of diabetes increased with the increase of RDW. In view of the convenience, noninvasiveness, and practicability of RDW detection, RDW might be considered for inclusion in the risk assessment of high-risk groups of diabetes.

## Figures and Tables

**Table 1:** Baseline characteristics of 16,971 Chinese adults across red blood cell distribution width quartile groups.

Variables	Red blood cell distribution width group (%)				*p* value
	Q1 ≤12.2	Q2 12.3~12.6	Q3 12.7~13.1	Q4 ≥13.2	
Sample, *n*	4,574	3,616	4,197	4,584	—
Sex, women (%)	39.9	38.7	38.0	47.9	<0.001
Age (y)	40.9 ± 12.2	42.7 ± 12.5	44.4 ± 12.9	45.4 ± 13.0	<0.001
Body mass index (kg/m^2^)	23.5 ± 3.08	23.9 ± 3.27	23.9 ± 3.3	23.6 ± 3.3	<0.001
Systolic blood pressure (mmHg)	118.6 ± 15.6	119.5 ± 15.5	119.96 ± 16.5	120.2 ± 16.4	<0.001
Diastolic blood pressure (mmHg)	74.3 ± 10.96	74.98 ± 10.9	75.3 ± 11.3	75.0 ± 11.3	<0.001
Total cholesterol (mmol/L)	4.9 ± 0.9	4.9 ± 0.9	4.96 ± 0.9	4.9 ± 0.9	<0.001
Triglyceride (mmol/L)^∗^	1.12 (0.79, 1.67)	1.16 (0.8, 1.7)	1.16 (0.81, 1.72)	1.09 (0.75, 1.61)	<0.001
High-density lipoprotein cholesterol (mmol/L)	1.4 ± 0.3	1.4 ± 0.4	1.4 ± 0.4	1.4 ± 0.4	<0.001
Low-density lipoprotein cholesterol (mmol/L)	2.9 ± 0.7	2.9 ± 0.7	2.9 ± 0.8	2.9 ± 0.8	0.02
Estimated glomerular filtration rate (mL/min per 1.73 m^2^)	108.5 ± 14.5	107.2 ± 14.3	106.1 ± 14.7	106.4 ± 15.4	<0.001
White blood cell count (10^9^/L)	6.2 ± 1.4	6.3 ± 1.5	6.4 ± 1.6	6.4 ± 1.7	<0.001
Alanine transferase (U/L)^∗^	17 (12, 26)	18 (13, 26)	17 (13, 25)	16 (11, 24)	<0.001
Aspartate transferase (U/L)	19.5 ± 8.5	19.7 ± 8.2	19.8 ± 8.3	19.3 ± 8.7	0.04
Red blood cell count (10^12^/L)	4.8 ± 0.4	4.9 ± 0.4	4.9 ± 0.4	4.8 ± 0.5	<0.001
Hemoglobin (g/L)	147.5 ± 13.4	147.1 ± 13.8	146.1 ± 14.1	137.9 ± 19.3	<0.001
Hematocrit (10^9^/L)	0.4 ± 0.04	0.4 ± 0.04	0.4 ± 0.04	0.4 ± 0.049	<0.001
High-sensitivity c-reactive protein (mg/L)^∗^	0.53 (0.26, 1.04)	0.6 (0.3, 1.18)	0.63 (0.32, 1.27)	0.61 (0.3, 1.34)	<0.001

Note: ^∗^abnormal distribution; data were presented as medium plus quartile range.

**Table 2:** Adjusted odds ratios and 95% confidence intervals for risk of diabetes across different red blood cell distribution width (RDW) quartile groups in 16,971 Chinese adults.

Model	Baseline RDW quartile groups (%)				Each percent of RDW	*p* trend
	Q1	Q2	Q3	Q4		
	≤12.2	12.3~12.6	12.7~13.1	≥13.2		
Sample	4,574	3,616	4,197	4,584	—	—
Case	619	532	716	836	—	—
Model 1	**1.00 (ref)**	1.03	1.14	1.18	1.13	0.001
		(0.92, 1.16)	(1.02, 1.27)	(1.06, 1.31)	(1.05, 1.21)	
Model 2	**1.00 (ref)**	0.97	1.08	1.17	1.13	0.001
		(0.86, 1.1)	(0.96, 1.2)	(1.05, 1.3)	(1.05, 1.22)	
Model 3	**1.00 (ref)**	0.99	1.1	1.22	1.17	<0.001
		(0.88, 1.12)	(0.98, 1.24)	(1.09, 1.38)	(1.08, 1.27)	
Model 4	**1.00 (ref)**	1.04	1.17	1.31	1.22	<0.001
		(0.91, 1.17)	(1.04, 1.32)	(1.16, 1.48)	(1.12, 1.33)	
Model 5	**1.00 (ref)**	0.99	1.11	1.2	1.16	<0.001
		(0.87, 1.12)	(0.99, 1.25)	(1.06, 1.36)	(1.06, 1.26)	

Note: model 1: adjusting for age (y) and sex. Model 2: adjusting variables in model 1 and further BMI (kg/m^2^), systolic blood pressure (mmHg), diastolic blood pressure (mmHg), total cholesterol (mmol/L), triglyceride (mmol/L), low-density lipoprotein cholesterol (mmol/L), high-density lipoprotein cholesterol (mmol/L), eGFR (mL/min per 1.73 m^2^), alanine transferase (U/L), and aspartate transferase (U/L). Model 3: adjusting variables in model 2 and further hemoglobin (g/L), hematocrit (10^9^/L), red blood cell count (10^12^/L), and white blood cell count (10^9^/L). Model 4: adjusting variables in model 3 and further high-sensitivity C-reactive protein (mg/L). Model 5: adjusting variables in model 4 and further fasting blood glucose (mmol/L) and glycated hemoglobin A1c (%).

**Table 3:** The adjusted hazardous ratios and 95% confidence interval for the risk of diabetes across red blood cell distribution width (RDW) quartile groups: sensitivity analyses.

		Baseline RDW quartile groups (%)				Each percent of RDW	*p* trend
		Q1 ≤12.2	Q2 12.3~12.6	Q3 12.7~13.1	Q4 ≥13.2		
Sensitivity-1	Sample	4,389	3,448	3,925	4,245	—	—
	Case	566	486	639	746	—	—
	Model	**1.00 (ref)**	1.04 (0.91, 1.18)	1.18 (1.05, 1.34)	1.32 (1.16, 1.5)	1.23 (1.13, 1.35)	<0.001
Sensitivity-2	Sample	3,574	2,768	3,128	3,383	—	—
	Case	439	349	483	539	—	—
	Model	**1.00 (ref)**	1.01 (0.87, 1.18)	1.21 (1.05, 1.39)	1.33 (1.14, 1.54)	1.24 (1.12, 1.38)	<0.001
Sensitivity-3	Sample	2,689	1,981	2,318	2,692	—	—
	Case	321	243	328	398	—	—
	Model	**1.00 (ref)**	1.01 (0.84, 1.22)	1.15 (0.97, 1.37)	1.33 (1.11, 1.6)	1.25 (1.1, 1.41)	<0.001
Sensitivity-4	Sample	4,564	3,606	4,173	4,546	—	—
	Case	617	528	712	830	—	—
	Model	**1.00 (ref)**	1.03 (0.97, 1.17)	1.17 (1.04, 1.32)	1.31 (1.16, 1.48)	1.23 (1.13, 1.34)	<0.001
Sensitivity-5	Sample	4,568	3,607	4,183	4,201	—	—
	Case	616	529	714	789	—	—
	Model	**1.00 (ref)**	1.03 (0.91, 1.17)	1.16 (1.03, 1.31)	1.31 (1.16, 1.48)	1.23 (1.13, 1.33)	<0.001

Note: Sensitivity-1: excluding participants whose age ≥ 65 y (*n* = 964). Sensitivity-2: excluding participants whose systolic blood pressure ≥ 130 mmHg or diastolic blood pressure ≥ 80 mmHg (*n* = 4,118). Sensitivity-3: excluding participants with overweight and obesity (*n* = 7,291). Sensitivity-4: excluding participants with decreased eGFR (*n* = 82). Sensitivity-5: excluding participants with anemia (*n* = 412). Adjusting for age (y), sex, BMI (kg/m2), systolic blood pressure (mmHg), diastolic blood pressure (mmHg), total cholesterol (mmol/L), triglyceride (mmol/L), low-density lipoprotein cholesterol (mmol/L), high-density lipoprotein cholesterol (mmol/L), eGFR (mL/min per 1.73 m2), alanine transferase (U/L), aspartate transferase (U/L), hemoglobin (g/L), hematocrit (109/L), red blood cell count (1012/L), white blood cell count (109/L), high-sensitivity C-reactive protein (mg/L), fasting blood glucose (mmol/L), and glycated hemoglobin A1c (%).

## Data Availability

The re-identified data and SAS code were available upon reasonable request (Renying Xu, email address: xurenying7465@126.com).

## Abbreviations

BMI: Body mass index
FBG: Fasting blood glucose
eGFR: Estimating glomerular filtration
HbA1c: Glycated hemoglobin A1c
RDW: Red blood cell distribution width.

## Supplementary Materials

Supplementary Materials Supplementary Table 1: comparison of baseline characteristics between participants in and out of the analysis. Supplementary Table 2: adjusted odds ratios and 95% confidence intervals for risk of diabetes across different red blood cell distribution width (RDW) quartile groups in 18,751 Chinese adults. Supplementary Table 3: the adjusted hazardous ratios and 95% confidence interval for the risk of diabetes across red blood cell distribution width (RDW) quartile groups in 16,971 Chinese adults: stratified by sex and age.
