# Supplementary material for: The Relationship between Red Blood Cell Distribution Width and Incident Diabetes in Chinese Adults: A Cohort Study
Source: J Diabetes Res. 2020 Feb 27;2020:1623247. doi: 10.1155/2020/1623247 (PMC7063217; doi:10.1155/2020/1623247)
Supplement: Supplementary Materials — Supplementary Table 1: comparison of baseline characteristics between participants in and out of the analysis. Supplementary Table 2: adjusted odds ratios and 95% confidence intervals for risk of diabetes across different red blood cell distribution width (RDW) quartile groups in 18,751 Chinese adults. Supplementary Table 3: the adjusted hazardous ratios and 95% confidence interval for the risk of diabetes across red blood cell distribution width (RDW) quartile groups in 16,971 Chinese adults: stratified by sex and age. [file 1623247.f1.pdf]

Supplementary Table 1. **Comparison of baseline characteristics between participants in and out of the analysis**

Variables	In the study	Out of the study	P value
Sample, n	16,971	37,863	--
Sex, women, %	41.3%	40.9%	0.08
Age, y	43.3±12.8	45.1±13.8	<0.001
FBG, mmol/L	5.0±0.5	5.3±0.2	<0.001
HbA1c, %	5.3±0.3	5.5±0.8	<0.001
RDW, %	12.8±0.9	1.28±0.9	0.84
BMI, kg/m^2	23.7±3.2	24.1±3.4	<0.001
SBP, mmHg	119.5±16.1	122.4±17.8	<0.001
DBP, mmHg	74.9±11.1	76.1±11.7	<0.001
TC, mmol/L	4.9±0.9	5.0±0.9	<0.001
TG, mmol/L*	1.13 (0.78, 1.67)	1.21 (0.83, 1.83)	<0.001
HDL, mmol/L	1.4±0.4	1.4±0.3	<0.001

LDL, mmol/L	2.9±0.8	2.9±0.8	0.17
eGFR, mL/min per 1.73 m^2	107.1±14.8	105.9±15.0	<0.001
WBC, 10^9/L	6.3±1.6	6.5±1.6	<0.001
ALT, U/L*	17 (12, 15)	18 (13, 27)	<0.001
AST, U/L	19.5±8.5	20.4±12.2	<0.001
RBC, 10^{12}/L	4.8±0.5	4.9±0.5	0.005
HB, g/L	144.5±16.0	145.4±16.0	<0.001
HCT, 10^9/L	0.44±0.04	0.44±0.04	<0.001
hs-CRP, mg/L	0.59 (0.29, 1.2)	0.65 (0.31, 1.35)	<0.001

Note:

1. Abbreviation: **FBG**, fasting blood glucose; **HbA1c**, glycated hemoglobin A1c; **RDW**, association between r ed 27 blood cell distribution width; **BMI**, body mass index; **SBP**, systolic blood pressure; **DBP**, diastolic blood pressure; **TC**, total cholesterol; **TG**, triglyceride; **HDL-C**, high density lipoprotein cholesterol; **LDL-C**, low density lipoprotein cholesterol; **eGFR**, estimated glomerular filtration rate; **WBC**, white blood cell count; **ALT**, alanine transferase; **AST**, aspartate transferase**;** **RBC**, red blood cell count; **HB**, hemoglobin; **HCT**, hematocrit; **hs-CRP**, high

sensitivity c-reactive protein.

2. *, abnormal distribution, data were presented as medium plus quartile range.

Supplementary Table 2. **Adjusted odd ratios and 95% confidence intervals for risk of diabetes across different red blood cell distribution width (RDW) quartile groups in 18,751 Chinese adults**

	Q1	Q2	Q3	Q4	Each percent of RDW	P trend
Sample	4,984	4,007	5,431	4,329	--	--
Case	667	580	919	777	--	--
Model	**1.00 (ref)**	1.01 (0.89,1.14)	1.19 (1.07,1.33)	1.28 (1.13,1.45)	1.2 (1.11,1.3)	<0.001

Note: Adjusting for age (y), sex, BMI (kg/m^2), systolic blood pressure (mmHg), diastolic blood pressure (mmHg), total cholesterol (mmol/L), triglyceride (mmol/L), low density lipoprotein cholesterol (mmol/L), high density lipoprotein cholesterol (mmol/L), eGFR (mL/min per 1.73 m^2), alanine transferase (U/L); aspartate transferase (U/L), hemoglobin (g/L), hematocrit (10^9/L), red blood cell count (10^{12}/L), white blood cell count (10^9/L), and high sensitivity C-reactive protein (mg/L), fasting blood glucose (mmol/L) and glycated hemoglobin A1c (%).

Supplementary Table 3. **The adjusted hazardous ratios and 95% confidence interval for the risk of diabetes across red blood cell distribution width (RDW) quartile groups in 16,971 Chinese adults: stratified by sex and age**

Group variables			Baseline RDW quartile groups (%)				Each percent of RDW	P trend
			Q1<br>≤12.2	Q2<br>12.3~12.6	Q3<br>12.7~13.1	Q4<br>≥13.2		
Sex	Men	Sample	2,750	2,217	2,603	2,386	--	--
		Case	393	327	470	460	--	--
		Model	**1.00 (ref)**	0.96<br>(0.82,1.13)	1.12<br>(0.96,1.3)	1.2<br>(1.02,1.41)	1.16<br>(1.04,1.3)	0.008
	Women	Sample	1,824	1,399	1,594	2,198	--	--
		Case	226	205	246	376	--	--
		Model	**1.00 (ref)**	1.17<br>(0.96,1.43)	1.3<br>(1.08,1.57)	1.54<br>(1.26,1.87)	1.35<br>(1.18,1.54)	<0.001

Age	<65 years	Sample	4,389	3,448	3,925	4,245	--	--
		Case	566	486	639	746	--	--
		Model	**1.00 (ref)**	1.04 (0.91,1.18)	1.18 (1.05,1.34)	1.32 (1.16,1.5)	1.23 (1.13,1.35)	<0.001
	≥65 years	Sample	185	168	272	339	--	--
		Case	53	46	77	90	--	--
		Model	**1.00 (ref)**	0.95 (0.6,1.49)	0.96 (0.63,1.47)	1.09 (0.72,1.67)	1.09 (0.82,1.46)	0.54

Note: Adjusting for age (y), sex, BMI (kg/m^2), systolic blood pressure (mmHg), diastolic blood pressure (mmHg), total cholesterol (mmol/L), triglyceride (mmol/L), low density lipoprotein cholesterol (mmol/L), high density lipoprotein cholesterol (mmol/L), eGFR (mL/min per 1.73 m^2), alanine transferase (U/L); aspartate transferase (U/L), hemoglobin (g/L), hematocrit (10^9/L), red blood cell count (10^{12}/L), white blood cell count (10^9/L), and high sensitivity C-reactive protein (mg/L), fasting blood glucose (mmol/L) and glycated hemoglobin A1c (%).
